# Usefulness of miRNA-338-3p in the diagnosis of pemphigus and its correlation with disease severity

**DOI:** 10.7717/peerj.5388

**Published:** 2018-08-03

**Authors:** Naiyu Lin, Qingxiu Liu, Menglei Wang, Qian Wang, Kang Zeng

**Affiliations:** Department of Dermatology, Nanfang Hospital, Southern Medical University, Guangzhou, Guangdong, China

**Keywords:** miR-338-3p, Bullae, Pemphigus, Target gene, Biomarker

## Abstract

**Background:**

Pemphigus is a common life-threatening, autoimmune bullous disease effecting both cutaneous and mucous membranes. Previous diagnosis of pemphigus is based on clinical presentations, histopathology, immunofluorescence and enzyme-linked immunosorbent assay. Furthermore, no laboratory parameters could be used to indicate disease severity. MicroRNAs are endogenous small RNAs, which could be used as diagnostic biomarkers for some autoimmune diseases. Previously, miR-338-3p has been proven significantly up-regulated in pemphigus patients.

**Methods:**

Pemphigus patients (including pemphigus vulgaris and pemphigus foliaceus) with active lesions and with remission, patients diagnosed as bullous pemphigoid and healthy volunteers were recruited, and miR-338-3p expression level was measured using reverse transcriptase-quantitative polymerase chain reaction (RT-qPCR). Active pemphigus patients accepting treatment were followed up for at least 2 weeks to investigate the expression change of miR-338-3p during treatment period. Target genes of miR-338-3p were screened through computer-aided algorithm and verified by RT-qPCR, Western blot and Luciferase activity assay.

**Results:**

MiR-338-3p was specifically increased in patients diagnosed as pemphigus with active lesions. The expression level of miR-338-3p gradually decreased after effective treatment. MiR-338-3p expression was independently correlated with disease severity defined by PDAI (Pemphigus Disease Area Index) or ABSIS (Autoimmune Bullous Skin Disorder Intensity Score) criteria. Up-regulation of miR-338-3p could significantly suppress RNF114 expression at mRNA and protein level in vitro.

**Discussion:**

MiR-338-3p could be used as a diagnostic biomarker of pemphigus in addition to other traditional methods. Up-regulation of MiR-338-3p was associated with more severe condition in pemphigus. RNF114 is the target gene of miR-338-3p, which probably participates in the regulation of disease activity of pemphigus.

## Introduction

Pemphigus is a rare life-threatening, autoimmune bullous disease involving mucous membranes and skin (Hammers & Stanley, 2016). The erosions of the skin and mucous membranes make pemphigus patients suffer from odynophagia, itching and loss of sleep, which will cause a sharp decrease of life quality. Moreover, due to the damage of skin barrier, the risk of infection was greatly increased (Leshem et al., 2014), which is a major complication causing high rate of mortality in these patients. Therefore, early and accurate diagnosis is extremely important for pemphigus patients. Nowadays, diagnosis of pemphigus is based upon clinical presentations, histopathology, immunofluorescence and enzyme-linked immunosorbent assay (ELISA) (Murrell et al., in press). However, histopathology and direct immunofluorescence (DIF) were cumbersome and time-consuming. ELISA method sometimes produces false-negative results (Belloni-Fortina et al., 2009). In addition, none of the above methods could be used to evaluate disease severity (Russo et al., 2017).

MicroRNAs (miRNAs) are endogenous small RNAs that play an important role in gene transcription and expression, involving in many biological functions such as cell proliferation, differentiation, metastasis and apoptosis. Recent years, miRNAs have been proven as key factors in regulating immune system development, normal immune function and autoimmunity (Alevizos & Illei, 2010). Furthermore, a few studies have also demonstrated that miRNAs can be used as diagnostic biomarkers in rheumatic diseases, such as systemic lupus erythematosus and systemic sclerosis due to its differential expression (Liu, Fan & Bai, 2015; Chouri et al., 2018). However, little is known about the miRNA expression in pemphigus.

Previous study from our department indicated that miR-338-3p was significantly up-regulated in peripheral blood monocular cells (PBMC) of patients with active pemphigus vulgaris (PV) compared with normal population (Wang et al., 2017). In the present study, we recruited more pemphigus patients to validate this differential expression. Furthermore, we followed up with those patients who had active pemphigus and accepted treatment for at least 2 weeks to investigate the changes of miR-338-3p expression before and after treatment. At the same time, we also evaluated the association between miR-338-3p expression level and disease severity of pemphigus. Finally, a direct target of miR-338-3p was found. The aim of the present study was to demonstrate the diagnostic role of miR-338-3p, as well as its correlation with disease severity in pemphigus and to find out possible target for future gene therapy research.

## Materials and Methods

### Population selection

According to the results of pre-experiment, the estimated effect size of miR-338-3p expression between pemphigus patients and normal population would be 0.56. The standard deviation (SD) was 0.62. We accept a *p* < 0.05 as acceptable and a study with 80% power. Using the following equation ${{{{\left( {{Z_{\rm{\alpha }}} + {Z_{1 - {\rm{\beta }}}}} \right)}^2} \times 2 \times {\sigma ^2}} \over {{\Delta ^2}}} $, we calculated that the sample size of the current study will be 20 (σ is the SD and Δ is the estimated effect size). Calculating for a 20% drop-out rate we need to complete at least 25 patients per group to be able to say with any degree of confidence whether a difference exists between groups (Bhalerao & Kadam, 2010).

A total of 42 patients were recruited at the Department of Dermatology, Nanfang Hospital, Southern Medical University to this study, of whom 35 patients were diagnosed as pemphigus including 32 PV patients and three pemphigus foliaceus (PF) patients. The remaining seven patients were diagnosed as bullous pemphigus (BP). The diagnostic criteria for PV/PF was defined as: (1) clinical presentation; (2) histopathology showing intraepidermal acantholysis in PV/PF; (3) DIF of perilesional skin showing IgG deposits at the surface of keratinocytes (Giurdanella et al., 2016). The diagnosis was made when (1) plus (2) or (3) were met.

Among 35 pemphigus patients, 30 patients had new blisters and had not been treated with immunosuppressants. While another five patients had achieved complete remission clinically, which was defined as no new blisters formation for more than 6 months. Patients diagnosed as paraneoplastic pemphigus were excluded in the present study because of possibly false-negative results. A total of 33 healthy subjects were recruited from the Physical Examination Center of Nanfang Hospital. The study protocol was approved by the Medical Ethics Committee of Nanfang Hospital (NFEC-2017-083). Written informed consent was obtained from all the participants.

### Clinical data

Patient-level data were obtained from the electronic medical record and laboratory databases. The electronic medical record consisted of patients age, gender, weight, diagnostic code, date of diagnosis, primary treatment, date of follow up. The laboratory data contained value of anti-Dsg-1 autoantibody, anti-Dsg-3 autoantibody, alanine aminotransferase (ALT), aspartate aminotransferase (AST), albumin (ALB), serum creatinine (CR), blood urea nitrogen (BUN), uric acid (UA), C reactive protein (CRP), white blood cell (WBC), neutrophil (NEU) and eosinophil (ESO).

PDAI and ABSIS were used to evaluate the severity of pemphigus in each patient according to the recent studies (Rahbar et al., 2014). Two doctors performed the scoring process independently to avoid the influence of subjective factors on the score results. Average score was obtained when the gap in score was less than 5. Disagreement was resolved by discussion with a third investigator. Severe pemphigus was defined as PDAI score >45 or ABSIS score >53 (Boulard et al., 2016). Relapse of pemphigus was defined as that patients had more than three new-onset skin lesions per month, which cannot heal in 1 week.

### Blood sample collection and preparation

A total of six ml venous blood was extracted from the pemphigus patients and healthy subjects. PBMC were isolated by Ficoll-Hypaque (TBD Science, Tianjing, Chian) density gradient centrifugation, lysed in TRIzol (Takara Bio, Kusatsu, Japan) and stored at −80 °C until use.

### Reverse transcriptase-quantitative polymerase chain reaction

A SYBR miRNA-assay kit (Takara Bio, Kusatsu, Japan) was used for the detection of miR-338-3p expression. cDNAs were prepared and stored at −20 °C until use. U6 gene was taken as an internal control for miRNAs and GAPDH gene was used as an internal control for mRNAs. All experiments were performed in triplicate and repeated once. The relative expression of miR-338-3p and its target gene was calculated using 2^−ΔΔCT^ method.

### Bioinformatics methods

The target genes of miR-338-3p predicted by computer-aided algorithms were obtained from miRwalk (http://129.206.7.150/search_mirnas/) and TargetScan (http://www.targetscan.org/vert_72/).

### Cell culture and transfection

Primary PBMCs from health people were isolated in RPMI 1640 (Gibco, Langley, OK, USA) medium supplemented with 10% fetal bovine serum (Gibco, Langley, OK, USA), 100 U/ml penicillin and 100 mg/ml streptomycin, cultured in humidified air at 37 °C with 5% CO_2_. Transfections were conducted for miR-338-3p mimics, miR-338-3p inhibitor and negative control (NC) using Lipofectamine™ 2000 (Invitrogen, Carlsbad, CA, USA) according to the manufacturer’s instructions. Following culture for a further 48 h, total RNA and cellular protein lysates were collected and used for reverse transcriptase-quantitative polymerase chain reaction (RT-qPCR) and western blot analysis, respectively.

### Western blot analysis

Expression of RNF114 protein was measured by Western blot analysis. Cell protein lysates were separated in 10% sodium dodecyl sulfate polyacrylamide gels, electrophoretically transferred to polyvinylidene difluoride membranes (Millipore, Burlington, MA, USA), and detected with anti-RNF114 antibody (GeneTex, Irvine, CA, USA). Protein loading was estimated using mouse anti-GAPDH monoclonal antibody.

### Assay of luciferase activity

The 3′UTR of RNF114 was amplified and cloned into the downstream of psiCHECK-2/Luciferase vector. Then the mutant 3′UTR of RNF114 (several nucleotides within the binding sites were mutant) was amplified using psiCHECK-2/Luciferase-RNF114 3′UTR as the template and cloned into the downstream of psiCHECK-2/Luciferase vector. For the luciferase reporter assay, the cells were co-transfected with miR-338-3p mimics or control and psiCHECK-2/Luciferase-RNF114 3′UTR or the mutant 3′UTR. The cells were lysed using Passive Lysis buffer 48 h after transfection. Luciferase intensity was measured by an Fluorescence Spectrophotometer (GloMax; Promega, Madison, WI, USA).

### Statistical analysis

All study data were stored in a standard EXCEL database. All the statistical analysis was performed using SPSS version 20.0 for windows (SPSS Inc., Chicago, IL, USA). Continuous variables were expressed as mean (*x*) ± SD. Categorical variables were expressed as frequencies and percentages (*n*, %). Baseline characteristics were analyzed by chi-square test or Fisher exact tests, if appropriate, and analysis of variance (ANOVA). Tukey’s multiple comparison test was used after the overall analysis in ANOVA. Diagnostic efficiency of miR-338-3p was analyzed by receiver operating characteristic (ROC) curve analysis. Repeated measurement data were analyzed by repeated ANOVA and generalized estimating equations (GEE). The value of *p* < 0.05 was considered statistically significant.

## Results

### MiR-338-3p is up-regulated specifically in patients with active pemphigus

**Figure 1:**
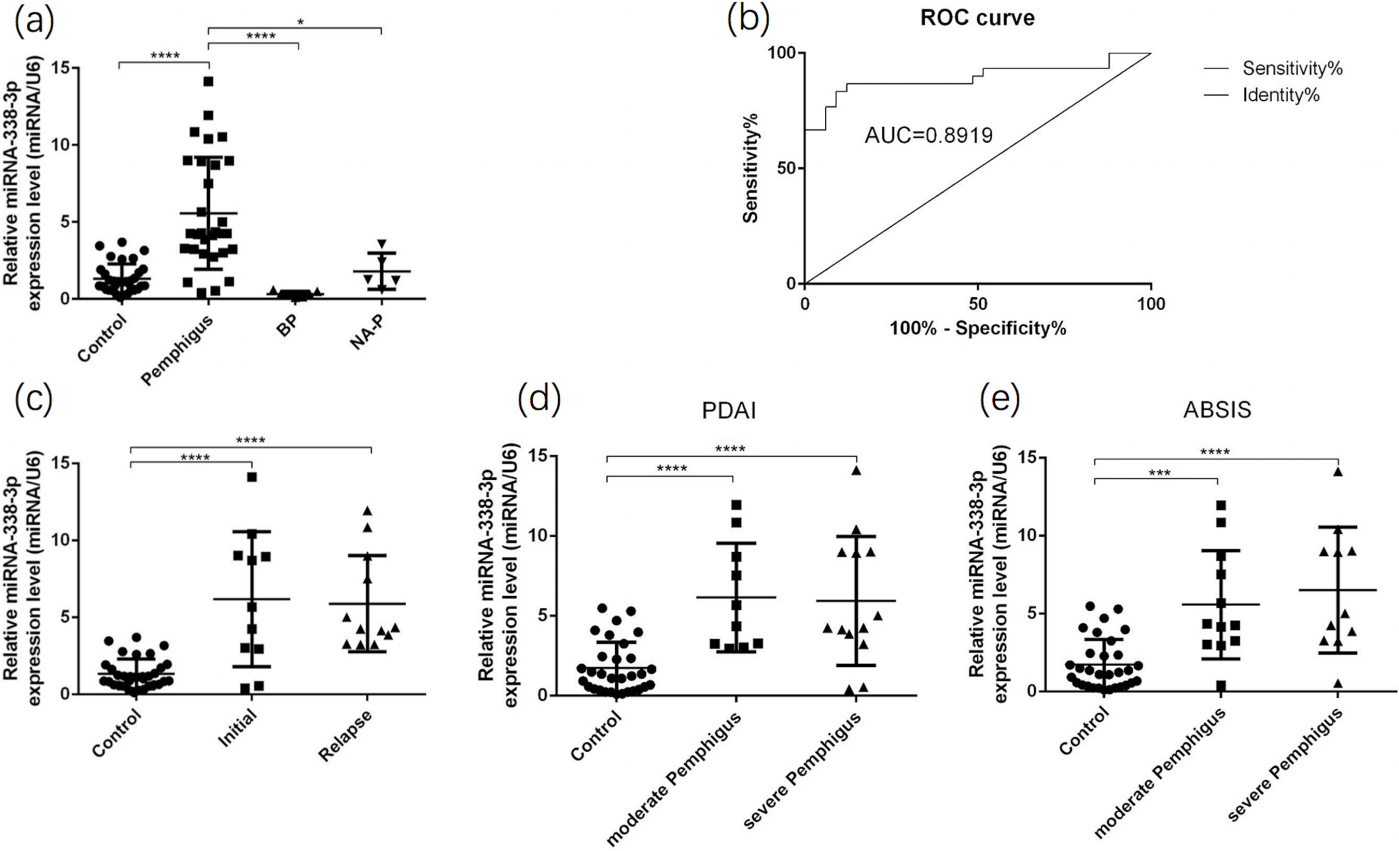
Diagnostic significance of miR-338-3p. (A) miR-338-3p expression levels between different groups; (B) ROC curve of miR-338-3p for the diagnosis of pemphigus; (C) miR-338-3p expression level in primary and recurrent patient; (D and E) miR-338-3p expression level in moderate and severe patient according to PDAI or ABSIS score. ANOVA was used in figure (A and C–E). Tukey’s multiple comparison test was used after the overall analysis in ANOVA. *p < 0.05, ***p < 0.001, ****p < 0.0001. Study sites: BP, bullous pemphigoid; NA-P, pemphigus with remission.

A total of 42 patients and 33 healthy subjects were included in this study. Baseline characteristics of all the participants were summarized in Table 1. Compared with the normal population, the expression of miR-338-3p was significantly increased in patients with active pemphigus. While, miR-338-3p expression was not increased in patients with BP and non-active pemphigus (Fig. 1A). Preliminary analysis based on the ROC analysis indicated a high predictive ability of miR-338-3p as pemphigus biomarker, with area under the curve (AUC) of 0.8919. The optimal cut off point was 2.676, which has a sensitivity of 86.67% and specificity of 87.88% (Fig. 1B). To further investigate the clinical significance of miR-338-3p, we divided pemphigus patients into subgroups. Firstly, there is no significant increase in miR-338-3p expression between patients with pemphigus as initial manifestation and those with relapse of pemphigus. Though, no significant difference on miR-338-3p expression was also identified between patients with moderate pemphigus and those with severe pemphigus, there is a tendency that the expression level of miR-338-3p is higher in patients with higher ABSIS scores (Figs. 1C–1E).

**Table 1:** Clinical characteristics of study population.

	NC	Pemphigus	BP	NA pemphigus	*P*-value
Number	33	30	7	5	–
Age	50.91 ± 11.84	59.77 ± 14.24	67.71 ± 19.65	43.20 ± 13.44	0.002
Sex (M:F)	18:15	16:14	3:4	0:5	0.139
MiR-338-3p level	1.31 ± 0.96	5.55 ± 3.64	0.31 ± 0.18	1.80 ± 1.18	<0.001

**Note:**

NC, normal control; BP, bullous pemphigoid; NA, not active.

### MiR-338-3p expression level is decreased during effective treatment

In order to verify that miR-338-3p could be used as a biomarker to demonstrate the effectiveness of treatment, 23 pemphigus patients were followed for at least 2 weeks after initial treatment with 14 patients being followed for 6 weeks. Within the nine patients lost to follow up, two of them refused to continue the study for personal reasons, two of them did not continue their therapy for economic issues, and five of them returned to their hometown to continue their treatment after partial remission.

**Figure 2:**
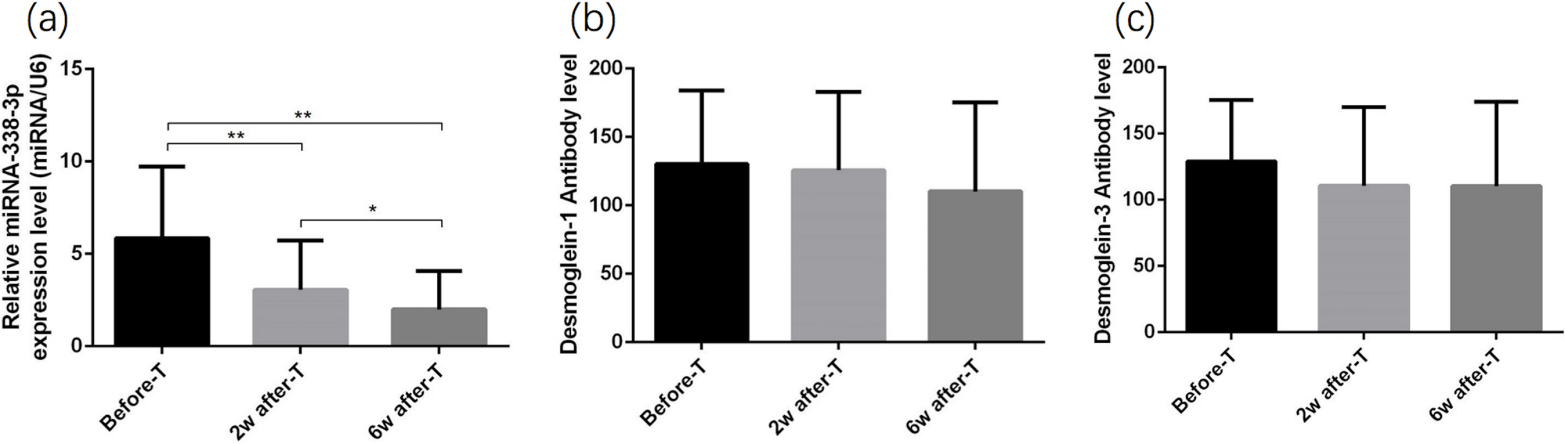
Expression change of miR-338-3p after effective treatment. (A) Differential expression level of miR-338-3p before treatment and 2 and 6 weeks after treatment; (B–C) differential expression level of anti-Dsg-1 and Dsg-3 antibodies before treatment and 2 and 6 weeks after treatment. Repeated ANOVA was used in figure (A–C). Tukey’s multiple comparison test was used after the overall analysis in ANOVA. *p < 0.05, **p < 0.01. Study sites: T, treatment.

All the patients’ conditions were considered improved based on the decrease in PDAI and ABSIS scores in spite of different therapy. The expression of miR-338-3p gradually decreased during effective treatment, which was significantly different between pre-therapy period and post-therapy period (Fig. 2A). However, the level of anti-Dsg-1 and anti-Dsg-3 antibodies, which play an important role in the pathogenesis of pemphigus did not present any significant difference among each period (Figs. 2B and 2C).

A total of nine patients were excluded from the repeated ANOVA measurement, which could not analyze missing values. In order to eliminate the possible selection bias when using repeated ANOVA, we conducted GEE to further investigate the correlation between miR-338-3p expression and effective treatment (Table 2). The expression of miR-338-3p significantly declined during effective treatment (*p* < 0.001), which was consistent with previous analysis. While, referring to anti-Dsg-1 and anti-Dsg-3 antibody, the level of anti-Dsg-1 antibody only showed significant difference 6 weeks after treatment and the level of anti-Dsg-3 antibody did not present significant decrease during effective therapy.

**Table 2:** Expression level of miR-338-3p, anti-Dsg-1 and anti-Dsg-3 antibody during treatment.

	miR-338-3p			Anti-Dsg-1 antibody			Anti-Dsg-3 antibody		
	Coefficient	SE	*p*-value	Coefficient	SE	*p*-value	Coefficient	SE	*p*-value
After 6 weeks	−3.895	0.6548	<0.001	−20.833	8.7781	0.018	−15.028	11.4126	0.188
After 2 weeks	−2.704	0.5125	<0.001	−4.54	5.1338	0.377	−16.91	9.6434	0.08
Before treatment	0^a^			0^a^			0^a^		

**Notes:**

SE, standard error.

a Represented the control group.

### MiR-338-3p is associated with disease severity defined by PDAI or ABSIS

From previous analysis, we considered that expression level of miR-338-3p might be associated with disease severity. In order to verify this hypothesis and to avoid the influence of other confounding factors, GEE was performed to evaluate the correlation between disease severity, defined by PDAI and ABSIS separately, and miR-338-3p expression (Table 3). In unadjusted analysis, the association between miR-338-3P and disease severity was significant in both criteria, as well as the level of anti-Dsg-1 and anti-Dsg-3 antibody. When adjusted for time, patients’ weight, age, sex, level of anti-Dsg-1 antibody, anti-Dsg-3 antibody, ALT, AST, ALB, CR, BUN, UA, CRP, WBC, NEU, ESO, miR-338-3p and anti-Dsg-3 antibody level was independently associated with disease severity. While, anti-Dsg-1 antibody was not associated with disease severity.

**Table 3:** Association between miR-338-3p, anti-Dsg-1 and anti-Dsg-3 antibody expression and PDAI or ABSIS.

	PDAI			ABSIS		
	Coefficient	SE	*p*-value	Coefficient	SE	*p*-value
**Unadjusted model**						
miR-338-3p	2.449	0.717	0.001	3.956	1.0268	<0.001
Anti-Dsg-1-antibody	0.132	0.0626	0.035	0.172	0.0703	0.014
Anti-Dsg-3 antibody	0.142	0.0288	<0.001	0.147	0.0782	0.06
**Adjusted model**						
miR-338-3p	1.035	0.371	0.005	3.81	0.9301	<0.001
Anti-Dsg1 antibody	0.1	0.0536	0.062	0.037	0.0904	0.685
Anti-Dsg3 antibody	0.099	0.031	0.001	0.105	0.0462	0.023

**Note:**

SE, standard error.

### MiR-338-3p modulates the expression of RNF114 gene and its associated protein

**Figure 3:**
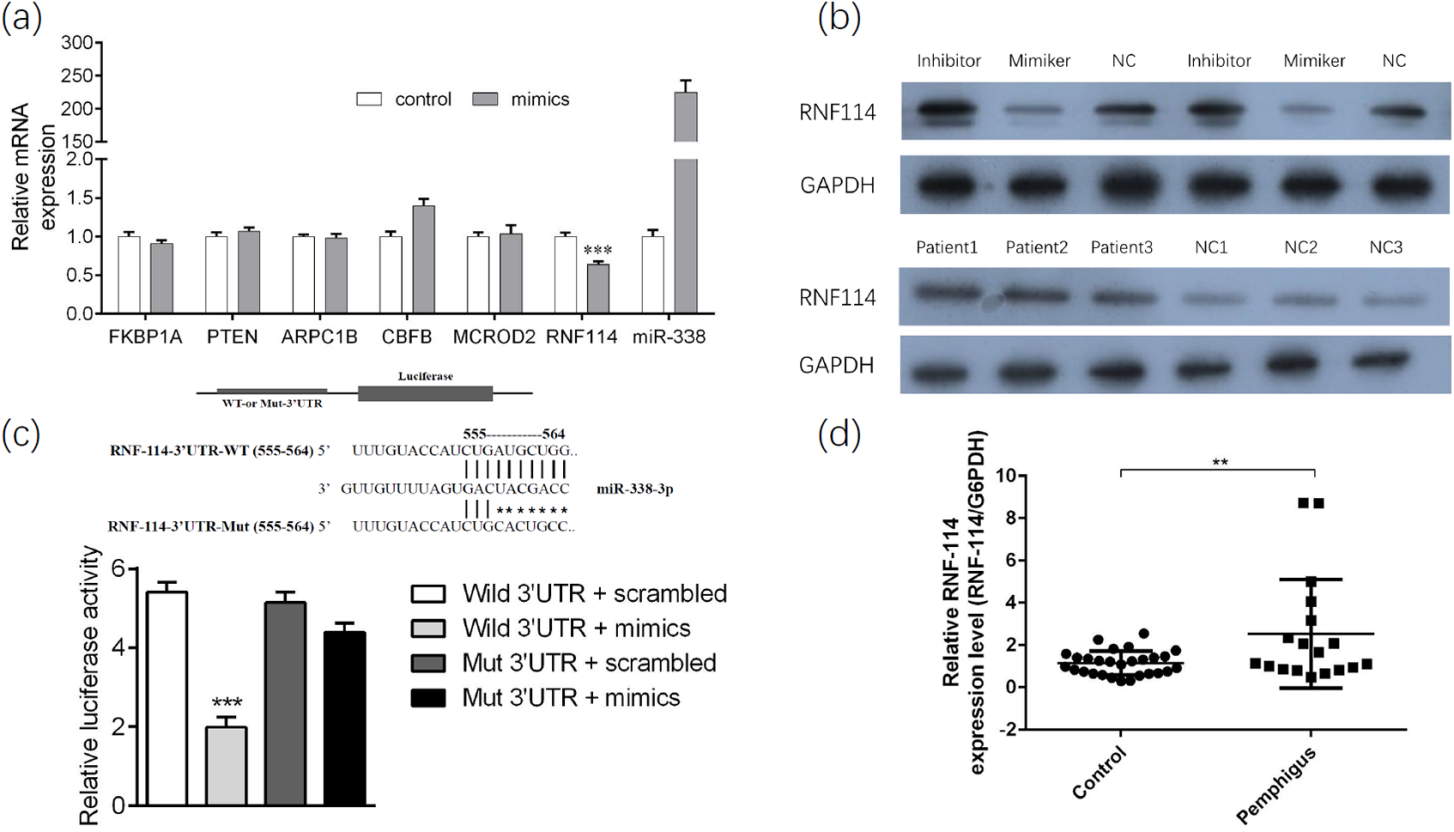
RNF114 was found as the target gene of miR-338-3p. (A) The PCR results of miR-338-3p and candidate target gene after transfection; (B) Western blot results of RNF114-associated protein after transfection in vitro (upper); Western blot results of RNF114-associated protein in vivo (lower); (C) dual-luciferase reporter gene assay results; (D) the PCR results of RNF114 of active pemphigus and health control group. **p < 0.01, ***p < 0.001. Study sites: UTR, untranslated regions; Mut, mutant; NC, negative control; GAPDH, glyceraldehyde 3-phosphate dehydrogenase.

To investigate the possible target gene of miR-338-3p in pemphigus, seven immune regulation related candidate genes were chosen through computer-aided algorithms. The expression level of miR-338-3p was up-regulated artificially through transfection. As a result, the expression of RNF114 gene was significantly down-regulated in both mRNA and protein level, which strongly indicated that RNF114 was the target gene of miR-338-3p (Figs. 3A and 3B). To confirm this hypothesis, the miR-338-3p binding sequences presented at 3′UTR of RNF114 mRNA (RNF114-3′UTR-WT) and mutant sequences (RNF114-3′UTR-mut) were subcloned into the downstream of the luciferase reporter gene in psiCHECK-2 vector. Comparing with RNF114-3′UTR-mut reporter group and NC group, relative luciferase activity of reporter of RNF114-3′UTR-WT was significantly decreased when co-transfected with miR-338-3p (Fig. 3C). However, contrary to the results in vitro, mRNA and protein level of RNF114 was up-regulated in pemphigus patients compared with normal population (Fig. 3B).

## Discussion

Pemphigus is a rare life-threatening autoimmune bullous disease, in which the anti-Dsg-1 and anti-Dsg-3 autoantibodies mediate the humoral immune response, causing deep erosions and blisters. Nowadays the diagnosis of pemphigus is mainly based on the assessment of clinical presentations, histopathologic results, direct and indirect immunofluorescence and ELISA. However, histopathology and DIF are cumbersome and time-consuming. ELISA method becomes more and more important in pemphigus diagnosis with high sensitivity and specificity. However, the wildly used method only detect Dsg-1 and Dsg-3 antibodies, which will sometimes cause false-negative results because of other pathogenic autoantibodies (Belloni-Fortina et al., 2009). In addition, anti-Dsg-3 antibodies have been detected in normal Egyptians (Saleh & El-Bahy, 2015). Recently, biochip immunofluorescence method has been developed in assistant diagnosis of pemphigus because of its simplicity of execution and low cost compared to ELISA method, as well as high sensitivity and specificity (Russo et al., 2014). However, BIOCHIP has not been broadly used in the world, which requires validation from large-sample-sized studies.

MicroRNAs play a significant role in immune response. They have been proven stable in vivo and easy to measure, which could be used as diagnostic biomarkers in certain disease (Zhang et al., 2013; Lindahl et al., 2018). To our knowledge, our study is the first research that investigates the diagnostic role of miR-338-3p in pemphigus. The results indicated that miR-338-3p is specifically overexpressed in active pemphigus patients compared to normal population with AUC of 0.8919, which can be used to diagnosis pemphigus with other diagnostic tools. Compared to ELISA and BIOCHIP method, the results of RT-qPCR could be used to define disease severity according to our studies. Additionally, the correlation between Dsg-1/3 and disease severity is controversial (Belloni-Fortina et al., 2009; Mortazavi et al., 2009) and the BIOCHIP could not provide quantitative value (Xuan, Yang & Murrell, 2018). However, due to the low incidence, we could not measure miR-338-3p in different subtypes of pemphigus, especially the paraneoplastic pemphigus, in which miR-338-3p expression level could be influenced by the tumor itself (Peng et al., 2014; Wang et al., 2015; Shan et al., 2015).

Previous researches have documented that early recognizing pemphigus patients’ response to initial therapy and modulating therapeutic regimens in time are important to control the active disease and to prevent patients from side effects of unnecessary immunosuppressants (Cholera & Chainani-Wu, 2016). Noteworthy, in our study, pemphigus patients were followed for at least 2 weeks, whose PBMC were extracted to measure miR-338-3p expression at 2 and 6 weeks after treatment. Our cohort demonstrated that miR-338-3p gradually decreased during effective treatment, which suggested that miR-338-3p can be used to evaluate patients’ response to initial therapy. While, anti-Dsg-3 antibody level showed no difference during treatment period and anti-Dsg-1 antibody level only presented significant decreasing 6 weeks after treatment. It is inconsistent with other studies, which demonstrated that decrease in anti-Dsg-1 antibody and anti-Dsg-3 antibody level is parallel with the clinical improvement in pemphigus patients (Barnadas et al., 2015). While this inconsistency probably due to the longer follow-up period in other studies. Our results provided an objective assessment of patients’ response to initial treatment compared to subjectively clinical assessment, which may be different between dermatologists. It is worth mentioning that treatment that all the patients received in our study was not unified and all the patients responded well to initial treatment. Therefore, further research is required to reveal expression level of miR-338-3p in patients who did not tolerate the initial therapy and the effect of certain drugs, on miR-338-3p expression.

Disease severity measurement is also important to guide physicians in when to reduce the dose of glucocorticoids in pemphigus. PDAI and ABSIS have been recognized as major criteria to evaluate the disease severity. However, these two score systems are too complicated to conduct in clinical settings. In our cohort, we have proven that miR-338-3p expression is independently associated with both PDAI and ABSIS scores in either unadjusted model or adjusted model, as well as anti-Dsg-3 antibody level. While anti-Dsg-1 antibody level did not correlated with disease severity. Thus, it is suggested that miR-338-3p expression level could be used to reflect disease severity in pemphigus, which is much more convenient than PDAI and ABSIS criteria. In our study, anti-Dsg-1 antibody level was not positively associated with disease severity, which probably resulted from that most of the patients in our cohort were diagnosed as PV instead of other types of pemphigus. Meanwhile, scores from PDAI and ABSIS are subjective, which could not truly reflect the disease severity of pemphigus. Further analyses are needed to validate this correlation and to reveal which part of disease activity can be best reflected by miR-338-3p.

The etiopathology of pemphigus is well understood with anti-desmoglein antibodies causing acantholysis, which result in deep erosions in mucous membrane and blisters in the skin (Furue & Kadono, 2017). However, the molecular mechanism underlying the production of antibodies and the regulation of humoral response have not been fully identified. For the purpose of understanding the underlying molecular mechanism of miR-338-3p in regulating immune response in pemphigus, we investigated its impact on various of gene expression. RNF114 has been previously proven to be a susceptible gene of psoriasis (Das et al., 2015). Recently, some studies also reported that it could stimulate T-cell activation and the C2H2 fragment might be its main functional site (Yang et al., 2014; Rodriguez et al., 2014). Our study proved that overexpression of miR-338-3p through transfection can reduce the expression of RNF114 mRNA and its related protein. Moreover, the interaction of miR-338-3p and mRNA of RNF114 in vitro was testified through luciferase reporter gene assay. However, in vivo, the expression level of RNF114-associated mRNA and protein was higher than that in healthy subjects. We suggested that RNF114 was up-regulated through other pathways in pemphigus patients as it could promote T-cell activation. Since miR-338-3p could suppress the function of RNF114, it would be overexpressed owing to the negative feedback. Further investigations are required to reveal the detailed underlying mechanism.

## Conclusion

In conclusion, the present study demonstrated that miR-338-3p expression was specifically up-regulated in active pemphigus patients. Thus, miR-338-3p could be used as a diagnostic biomarker of pemphigus in addition to other traditional methods. Moreover, the expression level of miR-338-3p significantly declined when the patient responded well to the initial treatment. Additionally, the expression of miR-338-3p was independently associated with disease severity defined by PDAI and ABSIS criteria. Finally, RNF114 was found as a direct target gene of miR-338-3p, which could promote T-cell activation and probably participates in the immune regulation of pemphigus.

## Supplemental Information

10.7717/peerj.5388/supp-1 Supplemental Information 1 Experimental and statistical results. Experimental and statistical results related to the article and the clinical data of the recruited patient.

10.7717/peerj.5388/supp-2 Supplemental Information 2 Comparison of expression level of miR-338-3p/anti Dsg-1 Antibody/anti Dsg-3 Antibody between different period using GEE. QIC: Quasi Likelihood under Independence Model Criterion; QICC: Corrected Quasi Likelihood under Independence Model Criterion a represented the control group.

10.7717/peerj.5388/supp-3 Supplemental Information 3 Results of association between miR-338-3p expression and PDAI or ABSIS of unadjusted/adjusted model. SE: standard error; ALT: alanine aminotransferase; AST: aspartate aminotransferase; ALB: Albumin; Cr: creatinine; BUN: blood urea nitrogen; UA: uric acid; CRP: C reactive protein; WBC: white blood cell; NEU: neutrophil; ESO: eosinophil.

10.7717/peerj.5388/supp-4 Supplemental Information 4 Uncropped blots of Figure 3.

## Additional Information and Declarations

### Data Availability

The following information was supplied regarding data availability: The raw data are provided in the Supplemental Files.
