# Supplementary material for: Usefulness of miRNA-338-3p in the diagnosis of pemphigus and its correlation with disease severity
Source: PeerJ. 2018 Aug 3;6:e5388. doi: 10.7717/peerj.5388 (PMC6078064; doi:10.7717/peerj.5388)
Supplement: Supplemental Information 2 — QIC: Quasi Likelihood under Independence Model Criterion; QICC: Corrected Quasi Likelihood under Independence Model Criterion a represented the control group. [file peerj-06-5388-s002.docx]

| TableS1-1 Comparison of expression level of miR-338-3p between different period using GEE | | | | | | | | | | | |
| --- | --- | --- | --- | --- | --- | --- | --- | --- | --- | --- | --- |
| Matrix Structure | Unstructured | | | | |  | Unstructured | | | | |
|  | Coefficient | SE | P value | 95%CI | |  | Coefficient | SE | P value | 95%CI | |
| Intercept | 6.02 | 0.75 | <0.001 | 4.54 | 7.498 | Intercept | 2.12 | 0.48 | <0.001 | 1.18 | 3.07 |
| After 6w | -3.90 | 0.65 | <0.001 | -5.18 | -2.61 | Before | 3.90 | 0.65 | <0.001 | 2.61 | 5.18 |
| After 2w | -2.70 | 0.51 | <0.001 | -3.71 | -1.7 | After 2w | 1.19 | 0.38 | 0.002 | 0.44 | 1.94 |
| Before | 0^a^ |  |  |  |  | After 6w | 0^a^ |  |  |  |  |
| Fit Index | QIC 548.03 | | QICC 548.64 | |  |  | QIC 548.03 | | QICC 548.64 | |  |
| QIC: Quasi Likelihood under Independence Model Criterion; QICC: Corrected Quasi Likelihood under Independence Model Criterion  a represented the control group | | | | | | | | | | | |

| TableS1-2 Comparison of expression level of anti-Dsg-1 Antibody between different period using GEE | | | | | | | | | | | | | |
| --- | --- | --- | --- | --- | --- | --- | --- | --- | --- | --- | --- | --- | --- |
| Matrix Structure | Autocorrelation | | | | | |  | Autocorrelation | | | | | |
|  | Coefficient | SE | P value | | 95%CI | |  | Coefficient | | SE | P value | 95%CI | |
| Intercept | 137.66 | 10.24 | <0.001 | | 117.59 | 157.73 | Intercept | 116.83 | | 13.20 | <0.001 | 90.97 | 142.69 |
| After 6w | -20.83 | 8.78 | 0.02 | | -38.04 | -3.63 | Before | 20.83 | | 8.78 | 0.02 | 3.63 | 38.04 |
| After 2w | -4.54 | 5.13 | 0.38 | | -14.60 | 5.52 | After 2w | 16.30 | | 8.78 | 0.06 | -0.92 | 33.50 |
| Before | 0^a^ |  |  | |  |  | After 6w | 0^a^ | |  |  |  |  |
| Fit Index | QIC 181606.65 | | | QICC 181607.16 | | |  | | QIC 181606.65 | | QICC 181607.16 | | |

| TableS1-3 Comparison of expression level of anti-Dsg-3 Antibody between different period using GEE | | | | | | | | | | | | | | |
| --- | --- | --- | --- | --- | --- | --- | --- | --- | --- | --- | --- | --- | --- | --- |
| Matrix Structure | Autocorrelation | | | | | | |  | | Autocorrelation | | | | |
|  | Coefficient | SE | P value | | 95%CI | | |  | | Coefficient | SE | P value | 95%CI | |
| Intercept | 118.62 | 12.56 | <0.001 | | 94.01 | 143.23 | | Intercept | | 103.60 | 13.75 | <0.001 | 76.64 | 130.54 |
| After 6w | -15.03 | 11.41 | 0.19 | | -37.40 | 7.34 | | Before | | 15.028 | 11.41 | 0.19 | -7.34 | 37.40 |
| After 2w | -16.91 | 9.64 | 0.08 | | -35.81 | 2.00 | | After 2w | | -1.88 | 9.10 | 0.84 | -19.71 | 15.95 |
| Before | 0^a^ |  |  | |  |  | | After 6w | | 0^a^ |  |  |  |  |
| Fit Index | QIC 226970.58 | | | QICC 226970.88 | | |  | | QIC 226970.58 | | | QICC 226970.88 | |  |
