# Supplementary material for: Usefulness of miRNA-338-3p in the diagnosis of pemphigus and its correlation with disease severity
Source: PeerJ. 2018 Aug 3;6:e5388. doi: 10.7717/peerj.5388 (PMC6078064; doi:10.7717/peerj.5388)
Supplement: Supplemental Information 3 — SE: standard error; ALT: alanine aminotransferase; AST: aspartate aminotransferase; ALB: Albumin; Cr: creatinine; BUN: blood urea nitrogen; UA: uric acid; CRP: C reactive protein; WBC: white blood cell; NEU: neutrophil; ESO: eosinophil. [file peerj-06-5388-s003.docx]

| TableS2-1 Results of association between miR-338-3p expression and PDAI or ABSIS of unadjusted model | | | | | | | | | | |
| --- | --- | --- | --- | --- | --- | --- | --- | --- | --- | --- |
|  | PDAI | | | | | ABSIS | | | | |
| Matrix Structure | Autocorrelation | | | | | Autocorrelation | | | | |
|  | Coefficient | SE | P value | 95% CI | | Coefficient | SE | P value | 95% CI | |
| miRNA | 2.45 | 0.72 | 0.001 | 1.04 | 3.85 | 3.956 | 1.03 | <0.001 | 1.94 | 5.97 |
| Fit Index | QIC | 27955.29 |  | QICC | 27953.84 | QIC | 47871.55 | QICC | | 47868.81 |
| Anti-Dsg-1 antibody | 0.13 | 0.06 | 0.035 | 0.01 | 0.26 | 0.172 | 0.07 | 0.014 | 0.04 | 0.31 |
| Fit Index | QIC | 27868.13 |  | QICC | 27865.58 | QIC | 49993.49 | QICC | | 49990.78 |
| Anti-Dsg-3 antibody | 0.16 | 0.04 | <0.001 | 0.07 | 0.25 | 0.184 | 0.05 | <0.001 | 0.08 | 0.28 |
| Fit Index | QIC | 24798.39 |  | QICC | 24798.39 | QIC | 46790.08 | QICC | | 46788.43 |
| Matrix Structure | Unstructured | | | | | Unstructured | | | | |
|  | Coefficient | SE | P value | 95% CI | | Coefficient | SE | P value | 95% CI | |
| miRNA | 2.45 | 0.65 | <0.001 | 1.17 | 3.73 | 4.79 | 0.84 | <0.001 | 3.16 | 6.43 |
| Fit Index | QIC | 27970.46 |  | QICC | 27969.65 | QIC | 53841.37 | QICC | | 53835.66 |
| Anti-Dsg-1 antibody | 0.09 | 0.06 | 0.118 | -0.02 | 0.21 | 0.16 | 0.08 | 0.043 | 0.01 | 0.32 |
| Fit Index | QIC | 27979.73 |  | QICC | 27978.21 | QIC | 53495.11 | QICC | | 53491.20 |
| Anti-Dsg-3 antibody | 0.14 | 0.03 | <0.001 | 0.09 | 0.20 | 0.15 | 0.08 | 0.06 | -0.01 | 0.30 |
| Fit Index | QIC | 24732.68 |  | QICC | 24734.11 | QIC | 46648.92 | QICC | | 46645.61 |

| Table S2-2 Results of association between miR-338-3p expression and PDAI or ABSIS of adjusted model | | | | | | | | | | |
| --- | --- | --- | --- | --- | --- | --- | --- | --- | --- | --- |
|  | PDAI | | | | | ABSIS | | | | |
| Matrix Structure | Autocorrelation | | | | | Autocorrelation | | | | |
|  | Coefficient | SE | P value | 95% CI | | Coefficient | SE | P value | 95% CI | |
| Intercept | 72.965 | 22.9784 | 0.001 | 27.928 | 118.002 | 52.769 | 34.3426 | 0.124 | -14.541 | 120.079 |
| [sex=0] | -11.502 | 4.7478 | 0.015 | -20.808 | -2.196 | -21.651 | 10.6463 | 0.042 | -42.518 | -0.785 |
| [sex=1] | 0^a^ | . | . | . | . | 0^a^ | . | . | . | . |
| age | -0.095 | 0.1791 | 0.595 | -0.446 | 0.256 | -0.012 | 0.3183 | 0.97 | -0.636 | 0.612 |
| miRNA | 1.035 | 0.371 | 0.005 | 0.308 | 1.762 | 3.81 | 0.9301 | <0.001 | 1.987 | 5.633 |
| Dsg-1 | 0.1 | 0.0536 | 0.062 | -0.005 | 0.205 | 0.037 | 0.0904 | 0.685 | -0.14 | 0.214 |
| Dsg-3 | 0.099 | 0.031 | 0.001 | 0.038 | 0.159 | 0.105 | 0.0462 | 0.023 | 0.015 | 0.196 |
| Weight | -0.564 | 0.0965 | <0.001 | -0.753 | -0.374 | -0.503 | 0.3049 | 0.099 | -1.101 | 0.094 |
| ALT | -0.261 | 0.0831 | 0.002 | -0.423 | -0.098 | -0.141 | 0.1465 | 0.336 | -0.428 | 0.146 |
| AST | 0.628 | 0.2482 | 0.011 | 0.141 | 1.114 | 0.592 | 0.3453 | 0.087 | -0.085 | 1.268 |
| ALB | -0.275 | 0.533 | 0.606 | -1.32 | 0.769 | 0.797 | 0.4171 | 0.056 | -0.021 | 1.614 |
| CR | -0.112 | 0.0931 | 0.23 | -0.294 | 0.071 | -0.141 | 0.2191 | 0.52 | -0.57 | 0.288 |
| BUN | 1.383 | 1.2921 | 0.285 | -1.15 | 3.915 | 1.073 | 1.513 | 0.478 | -1.893 | 4.038 |
| UA | -0.005 | 0.0259 | 0.857 | -0.056 | 0.046 | -0.036 | 0.0247 | 0.149 | -0.084 | 0.013 |
| CRP | 0.33 | 0.1309 | 0.012 | 0.073 | 0.586 | 0.217 | 0.1012 | 0.032 | 0.018 | 0.415 |
| WBC | -7.008 | 1.6786 | <0.001 | -10.298 | -3.718 | -8.777 | 3.7588 | 0.02 | -16.144 | -1.41 |
| NEU | 7.068 | 1.5528 | <0.001 | 4.025 | 10.112 | 9.053 | 3.7799 | 0.017 | 1.645 | 16.461 |
| ESO | -2.412 | 15.8524 | 0.879 | -33.483 | 28.658 | -5.229 | 11.3537 | 0.645 | -27.482 | 17.023 |
| Fit Index | QIC | 5647.528 |  | QICC | 5655.489 | QIC | 23725.418 |  | QICC | 23714.641 |
| Matrix Structure | Unstructured | | | | | Unstructured | | | | |
|  | Coefficient | SE | P value | 95% CI | | Coefficient | SE | P value | 95% CI | |
| Intercept | 86.668 | 34.6394 | 0.012 | 18.776 | 154.56 | -17.176 | 53.7028 | 0.749 | -122.431 | 88.08 |
| [sex=0] | -9.622 | 5.2363 | 0.066 | -19.885 | 0.64 | -23.796 | 12.1217 | 0.05 | -47.554 | -0.038 |
| [sex=1] | 0^a^ | . | . | . | . | 0^a^ | . | . | . | . |
| age | -0.185 | 0.2183 | 0.397 | -0.613 | 0.243 | 0.476 | 0.3406 | 0.162 | -0.192 | 1.144 |
| miRNA | 1.193 | 0.3558 | 0.001 | 0.496 | 1.891 | 4.674 | 0.9456 | <0.001 | 2.821 | 6.528 |
| Dsg-1 | 0.115 | 0.0406 | 0.004 | 0.036 | 0.195 | 0.029 | 0.1017 | 0.776 | -0.17 | 0.228 |
| Dsg-3 | 0.149 | 0.0278 | <0.001 | 0.095 | 0.204 | 0.147 | 0.0536 | 0.006 | 0.042 | 0.252 |
| weight | -0.465 | 0.1169 | <0.001 | -0.694 | -0.236 | -0.602 | 0.372 | 0.106 | -1.331 | 0.127 |
| ALT | -0.221 | 0.1037 | 0.033 | -0.424 | -0.018 | 0.187 | 0.1987 | 0.346 | -0.202 | 0.577 |
| AST | 0.649 | 0.2867 | 0.024 | 0.087 | 1.211 | 0.072 | 0.4566 | 0.874 | -0.823 | 0.967 |
| ALB | -0.512 | 0.718 | 0.476 | -1.919 | 0.895 | 1.674 | 0.6329 | 0.008 | 0.434 | 2.915 |
| CR | -0.189 | 0.1217 | 0.121 | -0.427 | 0.05 | 0.102 | 0.3083 | 0.742 | -0.503 | 0.706 |
| BUN | 1.703 | 0.8432 | 0.043 | 0.051 | 3.356 | 0.946 | 1.6643 | 0.57 | -2.316 | 4.208 |
| UA | -0.016 | 0.0294 | 0.594 | -0.073 | 0.042 | -0.042 | 0.0426 | 0.327 | -0.125 | 0.042 |
| CRP | 0.171 | 0.1632 | 0.295 | -0.149 | 0.491 | 0.132 | 0.1703 | 0.44 | -0.202 | 0.465 |
| WBC | -6.331 | 1.7661 | <0.001 | -9.792 | -2.869 | -7.452 | 5.1567 | 0.148 | -17.559 | 2.655 |
| NEU | 5.088 | 1.8205 | 0.005 | 1.52 | 8.656 | 7.016 | 5.2501 | 0.181 | -3.274 | 17.306 |
| ESO | -19.131 | 10.8351 | 0.077 | -40.368 | 2.105 | -4.492 | 19.0306 | 0.813 | -41.792 | 32.807 |
| Fit Index | QIC | 6326.339 |  | QICC | 6336.997 | QIC | 30896.86 |  | QICC | 30869.205 |
| SE: standard error; ALT: alanine aminotransferase; AST: aspartate aminotransferase; ALB: Albumin; Cr: creatinine; BUN: blood urea nitrogen; UA: uric acid; CRP: C reactive protein; WBC: white blood cell; NEU: neutrophil; ESO: eosinophil. | | | | | | | | | | |
