# Supplementary figures and images for: Usefulness of miRNA-338-3p in the diagnosis of pemphigus and its correlation with disease severity
Source: PeerJ. 2018 Aug 3;6:e5388. doi: 10.7717/peerj.5388 (PMC6078064; doi:10.7717/peerj.5388)

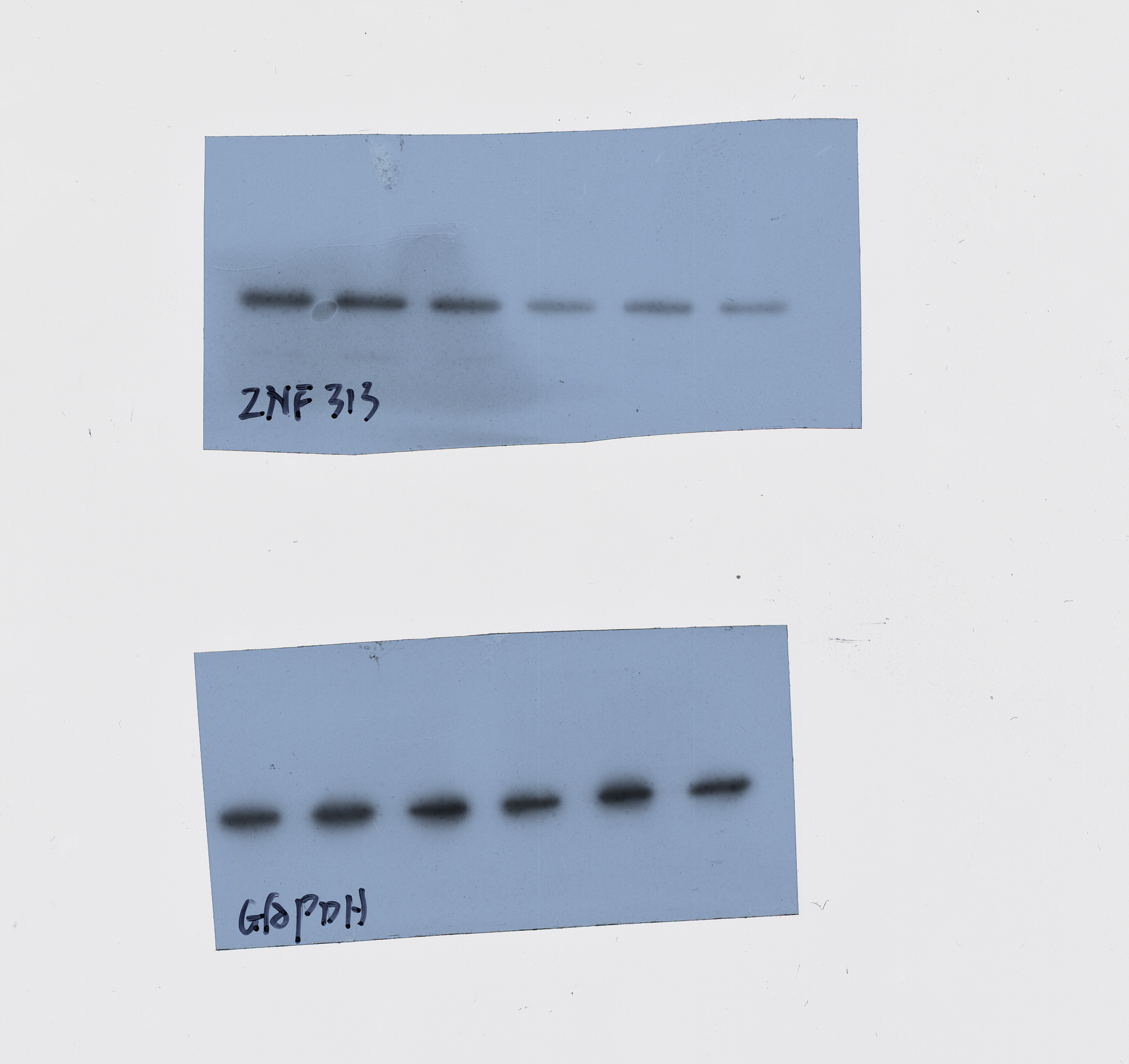

Supplement: Supplemental Information 4 [file peerj-06-5388-s004.zip › uncropped blots/1.tif]

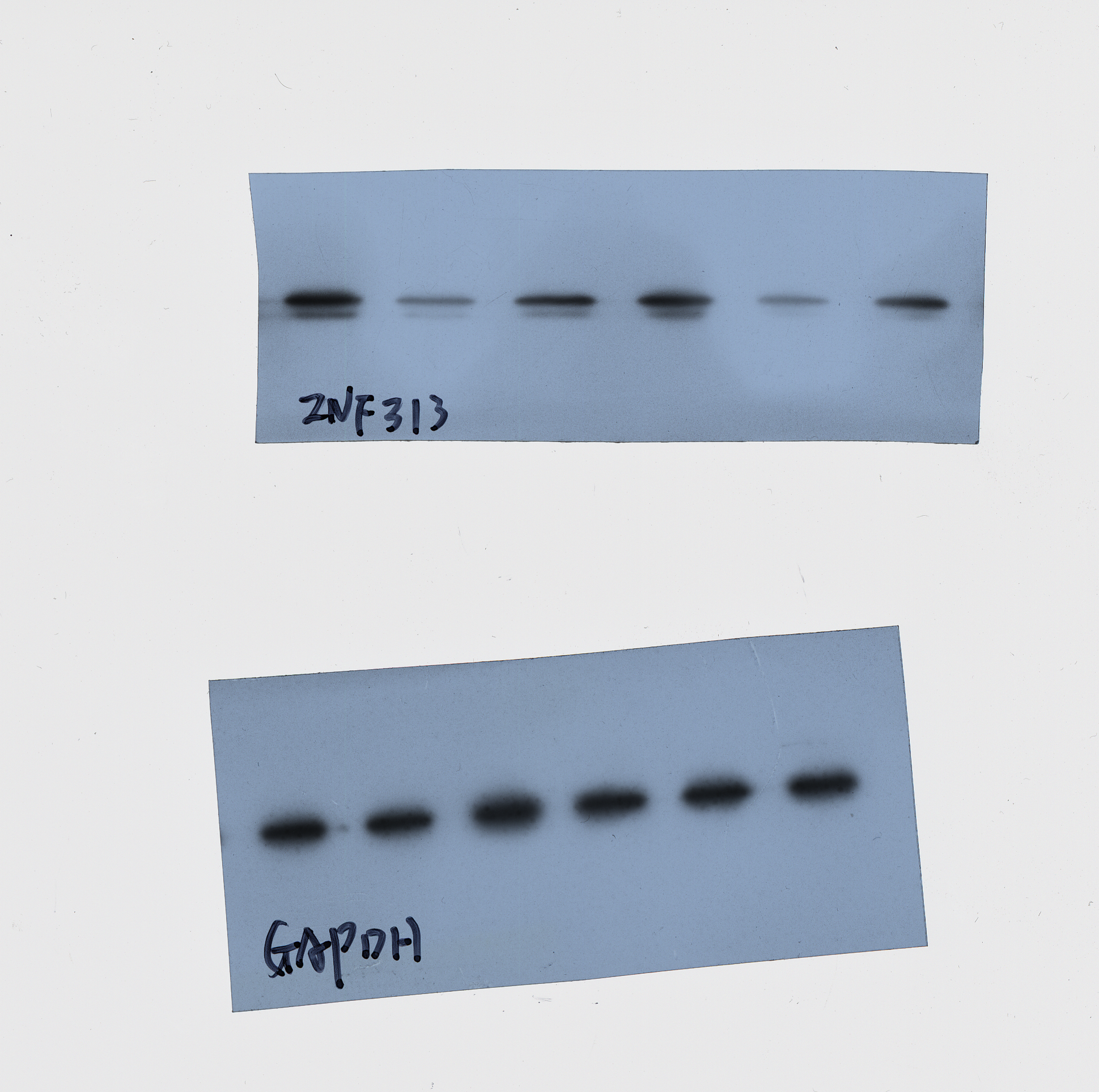

Supplement: Supplemental Information 4 [file peerj-06-5388-s004.zip › uncropped blots/2.tif]
